# Idiopathic spontaneous subacute subdural hematoma in a healthy young male: a case report

**DOI:** 10.11604/pamj.2026.53.36.46212

**Published:** 2026-01-27

**Authors:** Hajar Hamadi, Oumaima Monadi, Ayman Gallouli, Yassine Ait M’barek, Lamia Benantar, Khalid Aniba

**Affiliations:** 1 Department of Neurosurgery, Ibn Tofail Hospital, Mohammed VI^th^ University Hospital, Faculty of Medicine and Pharmacy in Marrakech, Cadi Ayyad University, Marrakech, Morocco

**Keywords:** Spontaneous subdural hematoma, subdural hematoma, subacute subdural hematoma, case report

## Abstract

Chronic and subacute subdural hematomas are a common pathology of the geriatric population. Idiopathic spontaneous chronic and subacute subdural hematoma are unusual, with only 22 reported cases in patients younger than 40 years old. We report in this article an unusual case of spontaneous subacute subdural hematoma in a 16-year-old healthy male presenting as a sudden onset of intracranial hypertension with an unremarkable physical and neurological examination. CT scan of the head confirmed the diagnosis, and the patient underwent surgical evacuation of the hematoma. Follow-up was unremarkable, and further blood tests were all within normal range. Sudden-onset intracranial hypertension in previously healthy patients should be further explored, and the possibility of a subdural hematoma should be considered in the differential diagnosis.

## Introduction

Subdural hematoma (SDH) is one of the most common neurosurgical pathologies, frequently seen in elderly patients after a minor head trauma [1-4]. Subdural hematoma refers to a blood collection located under the dural membrane. Thus, it is admitted that the presence of brain atrophy, increasing the space between the brain cortex and the calvarium, is a main risk factor for its occurrence in the geriatric population [3,5,6]. It is classified as acute, subacute, and chronic [3,5]. In the absence of trauma history, SDH is considered spontaneous and has been linked to several risk factors, including brain atrophy, systemic hypertension, coagulopathy, alcohol consumption, anticoagulant medications, and intracranial hypotension [1,7,8]. Idiopathic spontaneous subacute and chronic SDH in young adults with no apparent cause or risk factors are extremely unusual, with only 22 reported cases in the literature in patients younger than 40 years old. [1,8,9]. We report a case of idiopathic spontaneous subacute SDH in a 16-year-old healthy male presenting as a sudden onset of intracranial hypertension with an unremarkable physical and neurological examination as well as the imaging, surgical and follow-up features of his management.

## Patient and observation

**Patient information:** the patient is a 16-year-old male, with no personal or family medical records, presenting to the emergency department (ED) with a chief complaint of holocranial headaches.

**Clinical findings:** neurological examination of the patient showed a Glasgow coma scale of 15 and no neurological deficit, the rest of the neurological and physical examination was unremarkable.

**Timeline of the current episode:** the patient’s symptoms started two weeks prior to admission with holocranial headaches, which did not improve with medication. The symptoms persisted and were exacerbated by the occurrence of intermittent episodes of vomiting and blurred binocular vision and intermittent vomiting 6 days prior to his admission to the ED.

**Figure 1:**
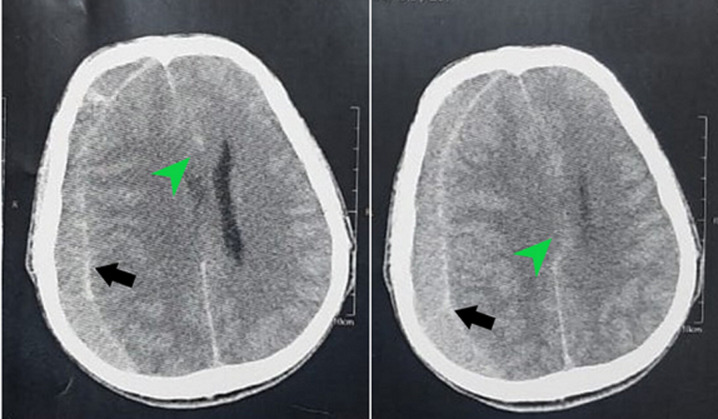
preoperative CT scan of the head in axial planes

**Diagnostic assessment:** emergency CT scan of the head showed an isodense right hemispheric subdural collection with mass effect on the right lateral ventricle and midline shift (Figure 1).

**Diagnosis:** imaging findings were consistent with a subacute subdural hematoma.

**Therapeutic interventions:** once the patient was deemed fit for surgery by the anesthesiology team, he was taken to the operating room. Evacuation of the hematoma was carried out under general anesthesia through a single Burr-hole, followed by profuse irrigation with a saline solution. A Jackson-Pratt drain system was placed in the subgaleal space. The patient’s symptoms improved after surgery.

**Follow-up and outcome of interventions:** the patient’s symptoms improved after surgery. A thorough interview was conducted with the patient and his family. They denied any history of trauma to the head, loss of consciousness or seizure. The patient had no history of frequent bleeding, no prior history of infection and no history of hematological disease. He also was not taking any chronic anticoagulants, antiplatelets or any other medications. Furthermore, he had no history of smoking, alcohol use or drug abuse (including recreational drug use and anabolic steroids), and his family history was negative for bleeding disorders, aneurysms and vascular malformations. During his hospital stay, additional blood investigations (blood smear, Factor VIII and XIII, and vWF cofactor) were performed, all of which were within normal limits. He was discharged on postoperative day 5 in stable condition and with fully resolved symptoms. The follow-up was unremarkable at 15 days, one month, 3 months and 6 months with no recurrence of the clinical symptoms.

**Patient perspective:** the patient was satisfied with the resolution of his headaches. The results of his blood tests and imaging findings were discussed with the parents. They were satisfied with the management and outcome as well as the detailed explanation regarding their son´s case.

**Informed consent:** it was obtained from the patient and his parents. All data were anonymized, and all identifying information was removed to guarantee the patient´s right to anonymity.

## Discussion

Spontaneous SDH is a fairly common pathology of the elderly, with enough data in the literature linking the incidence of chronic SDH to a myriad of factors [1,3]. Many authors have studied these risk factors, but the most commonly mentioned are advanced age with systemic hypertension, chronic alcoholism, use of blood thinners, and coagulopathies [1]. In the younger age group, this pathology is significantly less common and has its own risk factors, including hypertension, vascular malformations, neoplasia such as hematological malignancies causing thrombocytopenia, solid tumor dural metastases, infection, hypervitaminosis, coagulopathy, drug use and alcoholism [1,4,8,9]. There are other possible factors that could be related to spontaneous SDH and have been reported in multiple case reports, such as intracranial hypotension, steroid intake, and arachnoid cyst [1,10]. Spontaneous SDH is usually unilateral in young and middle-aged individuals with one or more of the predisposing factors previously mentioned [1,9]. Clinical manifestations include headache, gait and consciousness disturbances, personality changes, urinary incontinence, dementia, and worsening of pre-existing cognitive and psychiatric illnesses [1,3]. In most cases of spontaneous idiopathic SDH, the symptoms are vague and not specific, and the diagnosis is delayed until the severity of the symptoms worsens [3,9]. Despite the aforementioned statement, persistent headaches are a common revealing symptom [1,9]. A head CT scan is usually sufficient to confirm the diagnosis, and the treatment is similar to that of a subacute or chronic SDH using a burr hole trepanation to evacuate [5,6,10]. Follow-up is usually unremarkable with full recovery and regression of the symptoms [1,9].

The underlying pathophysiology of spontaneous idiopathic SDH is still not clearly understood, but several risk factors and hypotheses have been formulated [1,4,6,8]. It is believed that the structural and anatomical characteristics of the bridging veins make them more susceptible to rupture [1,8]. This hypothesis is backed up by the pathophysiology of spontaneous SDH in anabolic steroid users, which causes vascular remodeling and weakens the structure of the vein walls, increasing the susceptibility to bleeding [8,10]. Furthermore, some studies have proven the link between a rise of venous pressure and spontaneous SDH [1,3,8]. It was demonstrated that the sudden change in venous pressure, especially in the venules during forceable exhalation against a closed glottis - during a Valsalva maneuver, while blowing into a high-pressure instrument or a balloon, or heavy weightlifting - can lead to the rupture of these veins [1,6,8,10]. In our case, the patient had no underlying risk factors, no incident which would lead to a rise in the venous pressure, and no abnormalities on the biological and radiological investigation, which leaves us with no applicable explanation other than the fact that it was an idiopathic occurrence.

## Conclusion

Spontaneous idiopathic SDH in the young age group is a rare entity. The diagnosis is not always straightforward, as the clinical presentation is often insidious and misleading. It must be included as a differential diagnosis of persistent headache, unexplained neurological deterioration and de novo or worsening of psychiatric illness. An exhaustive etiological investigation is necessary to exclude possible causes before retaining the diagnosis. This entity remains fully unexplained by the existing hypotheses, and it is absolutely crucial to thoroughly investigate, especially in the young age group, before labeling a case as spontaneous and idiopathic SDH.
